# Diagrammatic Analysis of Nonhomogeneous Diffusion

**DOI:** 10.1155/2014/150826

**Published:** 2014-12-31

**Authors:** Julio A. Hernández

**Affiliations:** Sección Biofísica, Facultad de Ciencias, Universidad de la República, Iguá Esq. Mataojo, 11400 Montevideo, Uruguay

## Abstract

By virtue of its complexity, realistic approaches to describe diffusion in cellular media require the employment of computational methods. Among others, this type of studies has shown that the apparent diffusion coefficient of a macromolecular solute through a cytoplasmic-like medium exhibits a power-law dependence with the excluded volume. Power laws are ubiquitous findings in diverse systems, such as metabolic processes, population dynamics, and communication networks, and have been the object of many interpretative formal approaches. This study introduces a diagrammatic algorithm, inspired in previous ones employed to analyze multicyclic chemical systems, to derive expressions for nonhomogeneous diffusion coefficients and to study the effects of volume exclusion. A most noteworthy result of this work is that midsize diagrams of nonhomogeneous diffusion are already able to exhibit an approximate power-law dependence of the diffusion coefficient with the excluded volume. The employment of the diagrammatic method for the analysis of simple situations may thus prove useful to interpret some properties of larger network systems.

## 1. Introduction

The cellular compartment is a highly crowded medium of great structural heterogeneity [1–4]. Due to this complexity, the realistic approaches to represent diffusion in cellular media usually employ computational simulations [5–10]. Among other properties, this type of studies has shown that the apparent diffusion coefficient of a macromolecular solute through a cytoplasmic-like medium exhibits a power-law dependence with the excluded volume [8], in agreement with theoretical predictions from the study of mechanical models of polymers in solution [11–13] and consistent with experimental evidence [14–17]. Power laws are ubiquitous findings in many different types of processes, ranging from metabolism to communication networks, and have been the subject of many interpretative formal approaches (e.g., [18–20]). For a thorough revision and a historical perspective of this topic, the reader should see the articles in Newman et al. [21], and more recent surveys can be found in Clauset et al. [22] and Pinto et al. [23].

The general objective of this study is to contribute to the formal analysis of diffusion of solutes in cellular media. The specific purposes are to introduce a diagrammatic algorithm to derive explicit expressions of nonhomogeneous diffusion coefficients and to employ this method to study the dependence of the diffusion coefficient with the excluded volume. Since this work is not intended to contribute with complex realistic examples of nonhomogeneous diffusion but to introduce a formalism to interpret some basic aspects of this type of processes, the models analyzed here are relatively simple. Nevertheless, they already embody some properties characteristic of systems with a high degree of complexity, such as the aforementioned power-law dependence of the apparent diffusion coefficient with the excluded volume.

## 2. Diagrammatic Method for the Derivation of the Diffusion Coefficient of Solute Transport in Nonhomogeneous Media

The diagrammatic method was originally developed to analyze steady-state kinetics in chemical systems of intermediate complexity [24, 25] and was further employed to interpret diverse biochemical and biophysical processes, for instance, water and solute transport through biological membranes [26, 27]. As shown here, the method can be extended to obtain diffusion coefficients of steady-state diffusion in nonhomogeneous media. For this purpose, the nonhomogeneous medium is conceived as a network of transitions between selected positions or nodes, each one characterized by a specific concentration of the diffusing species. Discrete network approaches to represent nonhomogeneous processes of transport have been employed, for example, to understand the basic aspects of percolation [28]. The multicompartment representation adopted in this study permits expressing the transition of the solute between nodes via kinetic expressions. This type of strategies has been utilized, for instance, to understand the role of diffusion in brain processes [29] and to describe sarcomeric calcium movement [30].

**Figure 1:**
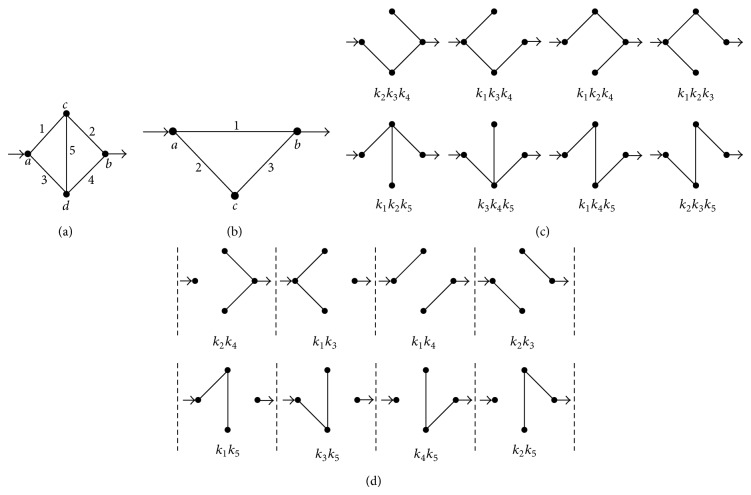
(a) and (b) Diagrams representing solute diffusion between positions “*a*” and “*b*”: a two-path model with two transitions each and an intermediate connection (a) and a model with two independent paths of different lengths (b). 1,2,…, 5 design transitions, governed by rate constants *k*_1_, *k*_2_,…, *k*_5_, respectively. For each transition, the two directions are governed by the same rate constant. (c) and (d) Individual paths and their appendages (c) and directional diagrams connecting positions “*a*” and “*b*” (d) for the model of (a). The corresponding products of rate constants are shown.

The flux of a permeating species through a membrane has been classically analyzed assuming the existence of a series of potential energy barriers. In this one-dimensional case, the kinetic formalism permits obtaining explicit expressions for the net flux in terms of the kinetic constants of jumping between neighbor positions in a rather straightforward fashion [31]. Similarly, the flux of a solute through a two- or three-dimensional nonhomogeneous medium can be conceived as mediated by transitions between positions separated by potential energy barriers. As mentioned, in these situations the derivation of explicit expressions for the solute fluxes may benefit from the employment of a simplifying algorithm, such as the diagram method proposed in this study. Instead of deriving general expressions, the procedure to obtain a kinetic expression for the nonhomogeneous diffusion coefficient is illustrated here employing the diagram shown in Figure 1(a). The basic assumption is that the diffusion of a solute between positions “*a*” and “*b*” only occurs via one or more specific paths that connect intermediate positions or nodes. As in chemical kinetics, the transitions connecting two neighbor positions are governed by rate constants. This work assumes that, for each transition, the rate constants in the two directions are equal. This assumption guarantees the accomplishment of the detailed balance condition in all of the cases. In steady state, the entrance flux into node *a* equals the exit flux at node *b*. The concentrations of the transported substance in these nodes (*C*_*a*_ and *C*_*b*_) and in the intermediate nodes (*C*_*c*_ and *C*_*d*_) are determined by the externally imposed steady-state flux (*J*) and by the rate constants (*k*_1_, *k*_2_,…). The rate constants (*k*'s) have dimensions LT^−1^.

For the model of Figure 1(a), characterized by different connected paths between nodes *a* and *b*, an adaptation of Hill's diagrammatic algorithm [25] may prove useful to derive the diffusion coefficient. In this context, the transition fluxes (*J*_1_, *J*_2_,…) can be defined as

(1a) $$\begin{array}{c} J_{1}=k_{1}\left(C_{a}-C_{c}\right);\;\;J_{2}=k_{2}\left(C_{c}-C_{b}\right);\\ J_{3}=k_{3}\left(C_{a}-C_{d}\right);\;\;J_{4}=k_{4}\left(C_{d}-C_{b}\right);\\ J_{5}=k_{5}\left(C_{d}-C_{c}\right). \end{array}$$

In steady state, the following relations between the transition fluxes and the steady-state flux *J* hold:

(1b) $$\begin{array}{c} J_{2}=J_{1}+J_{5};\;\;J_{4}=J_{3}-J_{5},\;\;J=J_{1}+J_{3}=J_{2}+J_{4}. \end{array}$$

From (1a) and (1b) and after some algebra, *J* can be expressed as

(1c) $$\begin{array}{c} J=\delta^{\prime }\left(C_{a}-C_{b}\right), \end{array}$$

where *δ*′ has dimensions LT^−1^ and is given by

(1d) δ′=k1k2(k3+k4+k5)+k3k4(k1+k2+k5)   +k5k1k4+k2k3×Δ−1,with  Δ=k1+k2k3+k4+k5k1+k2+k3+k4.

If *λ* is the distance between positions *a* and *b*, the overall diffusion coefficient *δ* may be defined as

(1e) $$\begin{array}{c} \delta =\delta^{\prime }\lambda . \end{array}$$

Equation (1d) can be directly obtained by applying the diagram method to the model of Figure 1(a), since it can be recognized that the numerator of *δ*′ is the sum of all the expressions corresponding to the trajectories between *a* and *b* and their appendages (Figure 1(c)), and the denominator (Δ) is the sum of all the directional diagrams of the model leading to nodes *a* and *b* (Figure 1(d)). This is a general property, ultimately a consequence of the accomplishment of the theorems of cyclic kinetic diagrams functioning in steady-state [25]. It can thus be employed to obtain the diffusion coefficient of any transport process represented by a discrete diagram. For the case of midsize models, such as the one of Figure 1(a), the determination of diffusion coefficients employing the diagram method results in more practical than explicit algebraic calculations.

If transition 5 does not exist (i.e., *k*_5_ = 0), the model of Figure 1(a) becomes a two-parallel-path model, and the diffusion coefficient ((1d)-(1e)) is given by

(2) δλ=k1k2k3+k4+k3k4k1+k2k1+k2k3+k4=k1k2k1+k2+k3k4k3+k4.

As can be seen, in this case *δ* is the sum of two diffusion coefficients, corresponding to each one of the independent (i.e., not interconnected) parallel paths. Equation (2) can be generalized to a diagram with an arbitrary number of independent paths connecting nodes *a* and *b*, where the overall diffusion coefficient corresponds to the sum of the diffusion coefficients of all the individual paths.

## 3. Effect of the Excluded Volume on the Diffusion Coefficient

**Figure 2:**
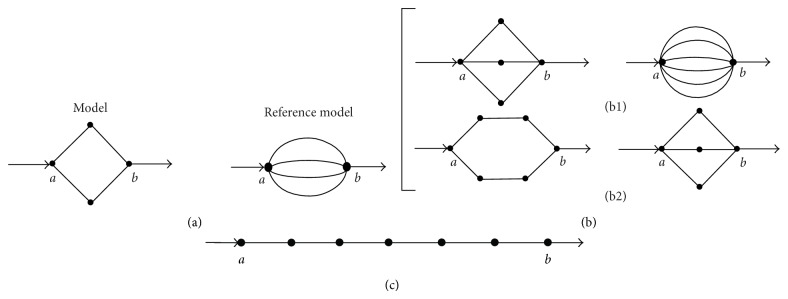
Diagrams of solute diffusion between “*a*” and “*b*”: two models with four transitions (a), multipath models with six transitions (b), and a single-path model with six transitions (c). In each case, the “reference models” are employed to derive expressions for the diffusion coefficients of the “models” (see main text).

As mentioned above, computer simulations of diffusion of small macromolecular tracers through complex nonhomogeneous media have empirically shown that the effect of the excluded volume is to determine a power-law dependence of the diffusion coefficient [8], a result that agrees well with predictions from theoretical studies of mechanical models [11–13] and with experimental evidence [14–17]. The present work now explores whether simple diagrams of nonhomogeneous solute diffusion already exhibit this property. For the case of the model shown in Figure 2(a), gradual suppression of the intermediate transitions determines increasing degrees of excluded volume. For this case, since there are four transitions, this procedure permits obtaining subdiagrams (SDs) for excluded volume (EV) values varying by units of one-fourth: 0, 1/4, 2/4, 3/4, and 4/4. For each one of these EV values, Table 5(a) shows all the corresponding resulting SDs. As can be easily concluded, the total number of SDs for each EV value can be obtained from the combinations formula as *n*!/[(*n* − *r*)!*r*!], where *n* is the total number of transitions in the original diagram and *r* the number of excluded transitions. In this simple case, the diffusion coefficient of each SD can be obtained either by direct algebraic calculus or by employing the diagram method illustrated above. Assuming that *λ* = 1 and *k*_1_ = *k*_2_ = *k*_3_ = *k*_4_ = *k* = 1 (in their corresponding units), Table 5(a) shows the values of *δ* obtained for each one of the resulting SDs. The average diffusion coefficient (*δ* ^*^) for each value of EV can therefore be determined. Thus, for instance, for EV = 2/4, *δ*_2/4_^*^ = [(2 × 0.5)+(4 × 0)]/6 = 1/6. For the case that EV = 0, *δ*_0_^*^ corresponds to the diffusion coefficient of the original model.  Table 1 lists the average diffusion coefficients calculated for all the EVs for the model (*δ* ^*^) and reference model (*δ*_R_^*^) shown in Figure 2(a). For the calculations, each one of the resulting subdiagrams can be treated independently. From inspection, it can readily be recognized that there are “equivalent” subdiagrams, a feature that simplifies determinations of the average coefficients.

**Figure 3:**
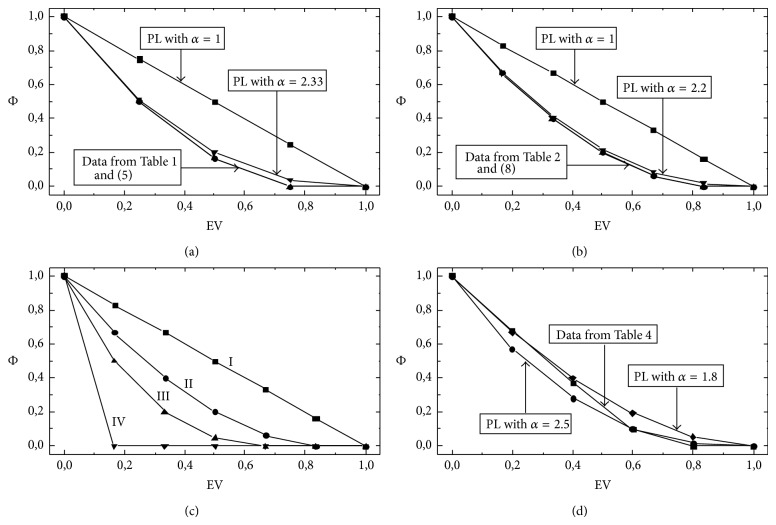
(a) and (b) Plots of the normalized diffusion coefficient (*Ф*) versus the excluded volume (EV) for the models of Figures 2(a) (a) and 2(b)(b1) (b).  Figure 3(a) employs the direct data for *Ф* contained in Table 1; (3) with *α* = 7/3 and *α* = 1; and (5). Figure 3(b) employs the direct data for *Ф* contained in Table 2; (3) with *α* = 11/5 and *α* = 1; and (8). (c) Similar to (a) and (b), but for the reference model (I) and model (II) of Figure 2(b)(b1); the model of Figure 2(b)(b2) (III) and the model of Figure 2(c) (IV). (d) Similar to (c), but for the model of Figure 1(a), employing direct data from Table 4, and (3) with *α* = 1.8 and 2.5. [*Ф* = *δ* ^*^/*δ*_0_^*^, where *δ* ^*^ is the average diffusion coefficient and *δ*_0_^*^ is *δ* ^*^ for EV = 0; PL: power law, (3); *λ* and all the rate constants equal 1].

The procedure just described can be employed to obtain average diffusion coefficients for any diagram, independently of its size, connectivity, and numerical values of the rate constants. For example, Table 5(b) contains all the SDs corresponding to the diagram of Figure 1(a). In this case, since the diagram contains five transitions, the EV values change as 0, 1/5,…, 5/5. Similarly to cyclical chemical diagrams, the degree of complexity increases with the number of intermediate nodes and connections in a nonlinear fashion [25]. For this reason, the diagram method can be applied rather straightforwardly to midsize diagrams of nonhomogeneous diffusion but becomes significantly involved for larger diagrams. Further examples of average diffusion coefficients obtained under the assumption that *λ* and all the rate constants equal 1 are shown in Tables 2, 3, and 4. Some of the data of these tables are employed to obtain the plots of Figure 3. Also, this figure includes approximations to the average diffusion coefficients (*δ*_app_^*^) obtained using the power law:

(3) $$\begin{array}{c} \frac{{\delta_{\text{app}}^{*}}^{}}{{\delta_{0}^{*}}^{}}={\left(1-\text{EV}\right)}^{\alpha }. \end{array}$$

For these approximations, *α* was calculated employing the procedures described below. Figure 3 reveals that, in the cases considered here, the average diffusion coefficients depend on the excluded volume approximately following a power-law dependence. In what follows, this work provides an interpretation of this finding.

## 4. Approximation by Power Laws to the EV-Dependence of the Diffusion Coefficient

The simplest models to consider are the ones that have noninterconnected paths of equal length. From the data of Table 1 it can be recognized that, for the model of Figure 2(a),

(4) ФФR=1−bEV, with  b=43,

where *Ф* and *Ф*_*R*_ are defined in the table.

Since *Ф*_*R*_ = 1 − EV, from (4) we obtain

(5) $$\begin{array}{c} Ф=\left(1-\text{EV}\right)\left(1-\frac{4}{3}\text{EV}\right)=1-\frac{7}{3}\text{EV}+\frac{4}{3}{\text{EV}}^{2}. \end{array}$$

Excluding higher-order terms in (5), we can approximate its logarithm by

(6) $$\begin{array}{c} \log\left(1-\frac{7}{3}\text{EV}\right)\sim -\frac{7}{3}\text{EV}. \end{array}$$

Taking logarithms of (4) and approximating them, we obtain

(7) $$\begin{array}{c} \log\left[{\left(1-\text{EV}\right)}^{\alpha }\right]=\alpha \log\left(1-\text{EV}\right)\sim -\alpha \text{EV}. \end{array}$$

From (6) and (7), *α* ~ 7/3.  Figure 3(a) shows the plots obtained for the model of Figure 2(a), employing the direct data for *Ф* of Table 1; (3) with *α* = 7/3 = 2.33 and *α* = 1; and (5). As can be seen, (3) with *α* = 7/3 provides a good approximation to the data.

Analogous reasoning permits obtaining, for the model of Figure 2(b)(b1), the following exact solution for the dependence of the average diffusion coefficient with EV:

(8) $$\begin{array}{c} Ф=1-a\text{EV}+b{\text{EV}}^{2}, \end{array}$$

where, in this case, *a* = 11/5 and *b* = 6/5.

Figure 3(b) shows the plots obtained for the model of Figure 2(b)(b1), employing the direct data for *Ф* contained in Table 2; (3) with *α* = *a* = 11/5 = 2.2 and *α* = 1; and (8). As can be seen, in this case the power law (3) provides a better approximation to the data than the model of Figure 2(a).

Equation (8) can be generalized to any model with an arbitrary number of independent two-transition paths. The generalization permits concluding that if *n* is the total number of transitions (i.e., *n* is in this case an odd number and *n*/2 is the total number of paths), *a* = (2*n* − 1)/(*n* − 1) and *b* = *n*/(*n* − 1). From these results, if *n* → *∞*, *a* → 2, the number of transitions per path.

For the case of the model shown in Figure 2(b)(b2), characterized by independent three-transition paths, the reference model is the one with the same total number of transitions (six) but with independent two-transition paths. Notice that this “reference model” is the “model” of Figure 2(b)(b1), analyzed above. This property and the linear dependence of *Ф*/*Ф*_*R*_ with EV (Table 3) permit deriving the following:

(9) $$\begin{array}{c} Ф=1-a\text{EV}+{b\text{EV}}^{2}-{c\text{EV}}^{3}, \end{array}$$

where, in this case, *a* = 37/10, *b* = 45/10, and *c* = 18/10.

Equation (9) is characteristic of all the models only possessing independent three-transition paths. In this case, *a* = (3*n* ^2^ − 6*n* + 2)/[(*n* − 1)(*n* − 2)]; *b* = [3*n*(*n* − 1)]/[(*n* − 1)(*n* − 2)]; and *c* = *n* ^2^/[(*n* − 1)(*n* − 2)]. Thus, if *n* → *∞*, *a* → 3, the number of transitions per path.  Figure 3(c) shows the direct results obtained for the average diffusion coefficients as functions of EV, assuming that all the rate constants equal 1, for four different six-transition models, connecting nodes *a* and *b* via the following independent paths: (1) six one-transition paths (reference model of Figure 2(b)(b1)); (2) three two-transition paths (model of Figure 2(b)(b1)); (3) two three-transition paths (model of Figure 2(b)(b2)); and (4) one six-transition path (model of Figure 2(c)). Figures 3(b) and 3(c) suggest that, for the case of models having a total of six transitions, the coefficient *α* of the approximated power law acquires values that fall within two limit ones, 1 and 6, according to the specific model structure.

From the above, generalization permits obtaining the following equation for networks made up of *n* total transitions and independent paths with the same number (*m*) of transitions per path:

(10a) Ф=1−a0EV1−a1EV1−a2EV⋯(1−am−1EV),with  ai=n(n−i), i=0,1,2,…,m−1.

From (10a), *a* is given by

(10b) $$\begin{array}{c} a=a_{0}+a_{1}+\cdots +a_{m-1}. \end{array}$$

Thus, for these networks, the coefficient (*α* ~ *a*) of the power law governing the dependence of the diffusion coefficient with the excluded volume depends in a rather simple, straightforward manner on the structural properties of the diagram.

Figure 1(b) shows the simplest model of nonhomogeneous diffusion possessing noninterconnected paths with different numbers of transitions. For this case, an exact equation for *Ф* can be derived taking as reference model the one with three independent single-transition paths (not shown). Following similar procedures as above, we can obtain the exact solution for the model of Figure 1(b):

(11) $$\begin{array}{c} Ф=1-\frac{3}{2}\text{EV}+\frac{1}{2}{\text{EV}}^{2}. \end{array}$$

In this case, (3) with *α* = 3/2 yields a good approximation to (11) (not shown). This value falls between the one of any model possessing only single-transition independent paths (*α* = 1) and the one of models consisting only of independent two-transition paths (*α* ≥ 2, cf. Figures 3(a) and 3(b)).

From the results obtained this far it can be concluded that, in order to exhibit power-law dependence of the diffusion coefficient with EV (i.e., with *α* > 1), the nonhomogeneous diffusion diagram must accomplish two necessary conditions: (i) it must possess more than one path and (ii) it must at least possess one path with more than one transition.

As an example of models with internal connections, this work considers the simple model shown in Figure 1(a). As above, the dependence of the average diffusion coefficient with the excluded volume can be determined from the analysis of the subdiagrams (Table 5(b)).  Figure 3(d) shows the corresponding plot (data for *Ф* ^*^ from Table 4) as well as those obtained employing (3) with *α* equal to 1.8 and 2.5. As can be concluded from the inspection of Figure 1(a) and Table 5(b), the subdiagrams of this model can be classified into two categories, according to whether they contain transition 5 or not. The two model categories can be analyzed independently to obtain the average diffusion coefficients (Table 4). Notice that the model obtained from the one of Figure 1(a) by excluding transition 5 is equivalent to the one of Figure 2(a), analyzed above (cf.  Table 1). Employing similar procedures as above, the following expression can be derived to account for the dependence of the average diffusion coefficient of the model and submodels containing transition 5 (*Ф*_5+_^*^) with the excluded volume:

(12) $$\begin{array}{c} {Ф}_{5+}^{*}=1-\frac{79}{36}\text{EV}+\frac{30}{36}{\text{EV}}^{2}. \end{array}$$

From inspection of Figure 3(d) it can be concluded that, for the case of the model shown in Figure 1(a), the dependence of the average diffusion coefficient with EV transits from a power-law approximation with *α* = 1.8 for low EV values to one with *α* = 2,5 for high EV values. A possible interpretation of this finding is that, at low EV values, the submodels with transition 5 dominate the EV-dependence (notice that *α* = 1.8 ~ 79/36) whereas the dependence is dominated by the submodels lacking transition 5 at higher EV values (*α* = 2.5 ~ 7/3, cf. (5)–(7)). Thus, models with inner connections exhibit complex dependence of the average diffusion coefficient with EV, described by power laws with variable coefficients.

## 5. Conclusions

This study has extended the diagram method of chemical kinetics to nonhomogeneous diffusion. For any network of paths connecting two nodes, the method permits obtaining the diffusion coefficient of steady-state solute transport. Theoretical studies of mechanical models of polymers in solution and computer simulations of nonhomogeneous diffusion have predicted power-law dependence of the apparent diffusion coefficient with the excluded volume. The diagrammatic analysis has been employed here to reveal that models relatively simple by comparison with realistic ones already exhibit this property. Thus, the diagrammatic analysis of diffusion models of intermediate complexity may provide a basis to interpret properties at a more complex level. This formalism may become helpful to confront evidence in contexts other than solute diffusion, such as the flow of information through communication networks or the propagation of epidemics in a population.

## Figures and Tables

**Table 1:** Average diffusion coefficients of the model (*δ* ^*^) and reference model (*δ*_R_^*^) shown in Figure 2(a). EV: excluded volume; ND: not defined.

EV	*δ* ^*^	Ф = *δ* ^*^/*δ*_0_^*^	*δ*_R_^*^	*Ф*_*R*_ = *δ*_R_^*^/*δ*_*R*,0_^*^	*Ф*/*Ф*_*R*_
0	1	1	4	1	1
1/4	1/2	1/2	3	3/4	2/3
2/4	1/6	1/6	2	2/4	1/3
3/4	0	0	1	1/4	0
4/4	0	0	0	0	ND

**Table 2:** Average diffusion coefficients of the model (*δ* ^*^) and reference model (*δ*_R_^*^) shown in Figure 2(b)(b1). EV and ND are as in Table 1.

EV	*δ* ^*^	Ф = *δ* ^*^/*δ*_0_^*^	*δ*_R_^*^	*Ф*_*R*_ = *δ*_R_^*^/*δ*_*R*,0_^*^	*Ф*/*Ф*_*R*_
0	1.5	1	6	1	1
1/6	1	2/3	5	5/6	4/5
2/6	6/10	2/5	4	4/6	3/5
3/6	3/10	1/5	3	3/6	2/5
4/6	1/10	1/15	2	2/6	1/5
5/6	0	0	1	1/6	0
6/6	0	0	0	0	ND

**Table 3:** Average diffusion coefficients of the model (*δ* ^*^) and reference model (*δ*_R_^*^) shown in Figure 2(b)(b2). EV and ND are as in Table 1.

EV	*δ* ^*^	Ф = *δ* ^*^/*δ*_0_^*^	*δ*_R_^*^	*Ф*_*R*_ = *δ*_R_^*^/*δ*_*R*,0_^*^	*Ф*/*Ф*_*R*_
0	2/3	1	1.5	1	1
1/6	1/3	1/2	1	2/3	3/4
2/6	2/15	1/5	6/10	2/5	2/4
3/6	1/30	1/20	3/10	1/5	1/4
4/6	0	0	1/10	1/15	0
5/6	0	0	0	0	ND
6/6	0	0	0	0	ND

**Table 4:** Average diffusion coefficients of the submodel containing transition 5 (*Ф*_5+_^*^), submodel lacking transition 5 (*Ф*_5−_^*^), and complete model (*Ф* ^*^) shown in Figure 1(a). EV is as in Table 1.

EV	*Ф*_5+_^*^	*Ф*_5−_^*^	*Ф* ^*^
0	1	0	1
1/5	3/5	1	17/25
2/5	5/18	1/2	11/30
3/5	0	1/6	1/10
4/5	0	0	0
5/5	0	0	0

**(a):** 

EV	Subdiagrams and diffusion coefficients					
0	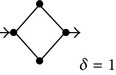					

1/4	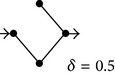	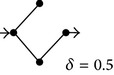	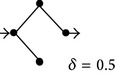	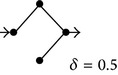		

2/4	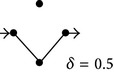	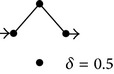	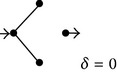	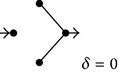	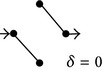	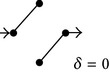

3/4	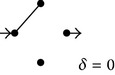	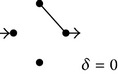	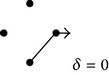	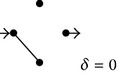		

4/4	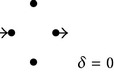					

**(b):** 

EV	Subdiagrams									
0	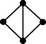									

1/5	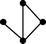	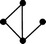	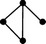	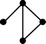	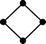					

2/5	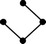	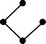	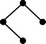	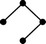	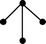	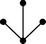	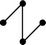	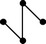	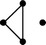	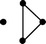

3/5	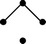	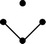	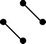	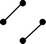	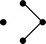	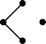	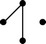	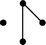	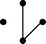	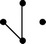

4/5	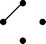	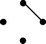	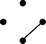	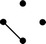	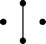					

5/5	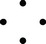									
